# COVID-19: Neurologic complications and management of neurological symptoms

**DOI:** 10.1192/j.eurpsy.2023.1716

**Published:** 2023-07-19

**Authors:** T. Jupe, B. Zenelaj, E. Myslimi, I. Giannopoulos

**Affiliations:** 1 Psychiatric Hospital of Attica, Athens, Greece; 2 National Center for Children Treatment and Rehabilitation; 3 Freelancer Psychiatrist, Tirane, Albania

## Abstract

**Introduction:**

Neurologic complications in patients with COVID-19 are common in hospitalized patients. More than 80 percent of hospitalized patients may have neurologic symptoms at some point during their disease course. Rates vary by geographical location and patient characteristics.

**Objectives:**

Τhe aim of this research is to evaluate the frequency of neurological complications in patients with covid-19.

**Methods:**

A literature review was made using the Pubmed Platform and the keywords: neurological symptoms, Covid-19 pandemic

**Results:**

Myalgias, headache, encephalopathy, and dizziness may be most common, occurring in approximately one-third of patients in China, Europe, and the United States. Neurologic symptoms such as dysgeusia or anosmia may be less common, but accurate ascertainment of symptoms may be limited in patients with severe cognitive or cardiorespiratory dysfunction. Stroke, movement disorders, motor and sensory deficits, ataxia, and seizures appear uncommon

**Conclusions:**

Reports of severe neurological involvement such as encephalitis, encephalopathy, status epilepticus, ischemic and hemorrhagic strokes and severe neuropathies (Guillain-Barré syndrome) in COVID-19 are increasing, which makes this problem particularly relevant to neurological critical care therapy.

**Disclosure of Interest:**

None Declared

